# Optimizing Topological Cascade Resilience Based on the Structure of Terrorist Networks

**DOI:** 10.1371/journal.pone.0013448

**Published:** 2010-11-10

**Authors:** Alexander Gutfraind

**Affiliations:** Center for Nonlinear Studies and T-5/D-6, Los Alamos National Laboratory, Los Alamos, New Mexico, United States of America; Indiana University, United States of America

## Abstract

Complex socioeconomic networks such as information, finance and even terrorist networks need resilience to cascades - to prevent the failure of a single node from causing a far-reaching domino effect. We show that terrorist and guerrilla networks are uniquely cascade-resilient while maintaining high efficiency, but they become more vulnerable beyond a certain threshold. We also introduce an optimization method for constructing networks with high passive cascade resilience. The optimal networks are found to be based on cells, where each cell has a star topology. Counterintuitively, we find that there are conditions where networks should not be modified to stop cascades because doing so would come at a disproportionate loss of efficiency. Implementation of these findings can lead to more cascade-resilient networks in many diverse areas.

## Introduction

Cascades are ubiquitous in complex networks and they have inspired much research in modeling, prediction and mitigation [1]–[10]. For example, since many infectious diseases spread over contact networks a single carrier might infect other individuals with whom she interacts. The infection might then propagate widely through the network, leading to an epidemic. Even if no lives are lost, recovery may require both prolonged hospitalizations and expensive treatments. Similar cascade phenomena are found in other domains such as power distribution systems [11]–[13], computer networks such as ad-hoc wireless networks [7], financial markets [14], [15] and socio-economic systems [16]. A particularly interesting class are “dark” or clandestine social networks, such as terrorist networks, guerrilla groups [17], espionage and crime rings [18], [19]. In such networks if one of the nodes (i.e. individuals) is captured by law enforcement agencies, he may betray all the nodes connected to him leading to their likely capture.

**Figure 1:**
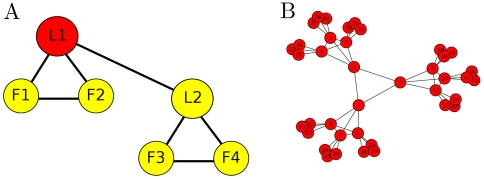
The French World-War II underground network *Francs-tireurs et Partisans* (FTP) reconstructed by the author based on the account in [**20**]. Its organizational unit was the combat group (A). In an idealized case, nor always followed, this was divided into two “teams” of three fighters, where leader L1 was in overall command and in command of team 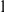. His lieutenant, L2, led team 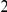 and assumed overall command if L1 was captured. The small degree of the nodes ensured that the capture of any one node did not risk the exposure of a significant fraction of the organization. Each “group” is in a command hierarchy (B) where 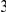 groups (bottom-level nodes) made a “section”, 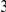 sections made a “company”, and finally 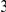 companies made a “battalion”.

Dark networks are therefore designed to operate in conditions of intense cascade pressure. As such they might serve as useful prototypes of networks that are cascade-resilient because of their connectivity structure (topology) alone. Their nodes are often placed in well-defined cells - closely-connected subnetworks with only sparse connections to the outside (for an example from World War II see Fig. 1) [20]. The advantages of cells are thought to be that the risk from the capture of any person is mostly limited to his or her cell mates, thereby protecting the rest of the network [21], [22]. Modern terrorist groups retain this cellular structure, but increasingly use networks made of components with no connections between them, thus caging cascades within each component [23]–[25].

To represent networks from different domains, this paper will use simple unweighted graphs. This approach offers simplicity and can employ tools from the well-developed field of graph theory. A simplification is also unavoidable given the lack of data on networks, especially on dark networks where only the connectivity is known, if that. Ultimately through, models of networks, especially dark networks must consider their evolving nature, fuzzy boundaries and multiplicities of node classes and diverse relationships.

Fortunately, the loss of information involved in representing networks as simple rather than as weighted graphs could be evaluated. In the File S1, we consider two unusually rich data sets where the edges could be assigned weights. We find that the error in using simple graphs has no systematic bias and is usually small.

### Evaluating Cascade Resilience of Networks

Our preliminary task is to compare the cascade resilience of networks from different domains. We will see that dark networks are indeed more successful in the presence of cascades than other complex networks. Their success stems not from cascade resilience alone but from balancing resilience with efficiency (a measure of their ability to serve their mission).

We will consider a particular type of cascade resilience and a particular definition of efficiency. For resilience we will use a probabifolistic process known as “SIR” (susceptible-infected-recovered). In SIR any failed (captured) node leads to the failure of each neighboring node independently with probability 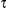 [26]. Using the SIR model, resilience 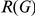 could be defined as the average fraction of the network that does not fail in the cascade. Efficiency 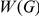 is also a function of the connectivity structure, and could be defined based on the distances between all pairs of nodes in the graph (see the Methods section for exact expressions.)

Observe that the most cascade-resilient network is the network with no edges (hence no cascades can propagate), but it is also the least efficient kind of network. It is expected that resilience and efficiency will be in opposition, requiring trade-offs. Just as disconnected networks are resilient and inefficient, highly-efficient networks such as densely-connected graphs are likely to have low resilience (for a historic example see [27].)

Define the overall “fitness”, 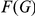, of a network by aggregating resilience and efficiency through a weight parameter 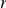:
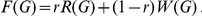
The parameter 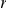 depends on the application and represents the cost of restoring the network after a cascade - from light (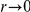) to catastrophic (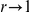). It is possible to include in fitness other metrics such as construction cost.

We will compare the fitnesses of several complex networks, including communication, infrastructure and scientific networks to the fitnesses of dark networks. The class of dark networks will be represented by three networks: the 9/11, 11M and FTP networks. The 9/11 network links the group of individuals who were directly involved in the September 11, 2001 attacks on New York and Washington, DC [28]. Similarly the 11M network links those responsible for the March 11, 2004 train attacks in Madrid [23]. Both 9/11 and 11M were constructed from press reports of the attacks. Edges in those networks connect two individuals who worked with each other in the plots [23], [28]. The FTP network is an underground group from World War II (Fig. 1), whose network was constructed by the author from a historical account [20].

**Figure 2:**
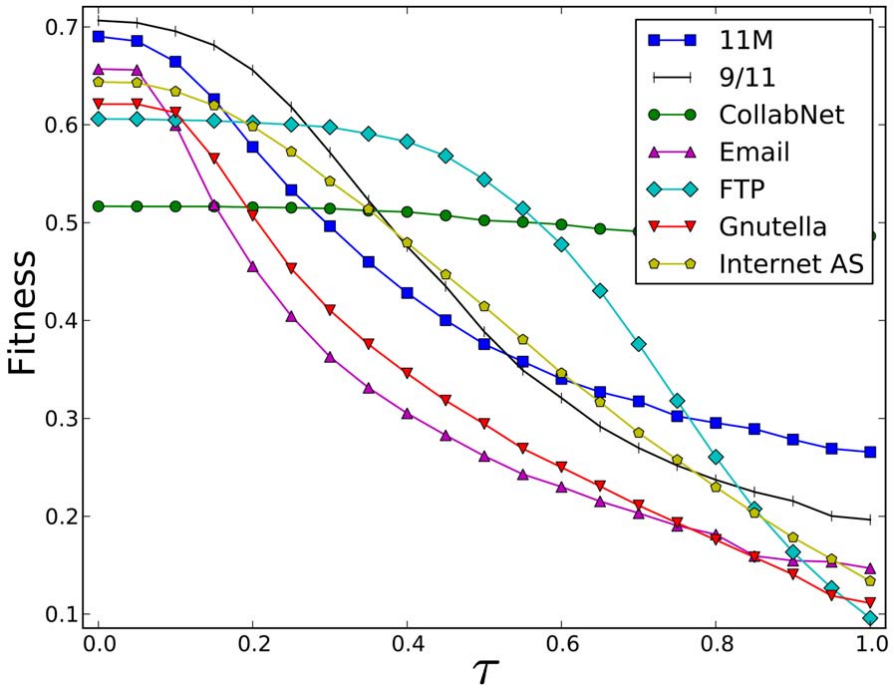
Fitnesses of various networks at 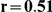 and various values of 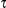. 11M is the network responsible for the March 11, 2004 attacks in Madrid (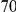 nodes, 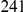 edges). 9/11 [28] is the network responsible for the 9/11 attacks (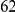 nodes, 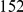 edges). CollabNet [29] is a scientific co-authorship network in the area of network science (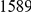 nodes, 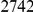 edges). E-Mail [37] is a university's e-mail contact network, showing its organizational structure (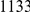 nodes, 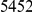 edges). FTP is the network in Fig. 1 (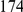 nodes, 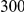 edges). Gnutella [38], [39] is a snapshot of the peer-to-peer network (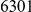 nodes, 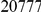 edges). Internet AS [40] is a snapshot of the Internet at the autonomous system level (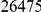 nodes, 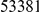 edges). Except for 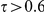 dark networks (11M, 9/11 and FTP) attain the highest fitness.

Figure 2 shows that the dark networks attain the highest fitness values of all networks, except for extreme levels of cascade risk (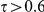). This is to be expected: only 11M, 9/11, and the FTP networks have been designed with cascade resilience as a significant criterion - a property that makes them useful case studies. For high cascade risks (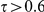) the CollabNet network exceeds the fitnesses of the dark networks. CollabNet was drawn by linking scientists who co-authored a paper in the area of network science [29]. It achieved high fitness because it is partitioned into research groups that have no publications with outside scientists. Like some terrorist networks, it is separated into entirely disconnected cells.

The 9/11 and the 11M networks are very successful for low values of 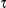 (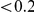), but then rapidly deteriorate because of a jump in the extent of cascades - the so-called percolation transition [30]. Past this threshold, cascades start affecting a large fraction of the network, resilience collapses and the fitness declines rapidly. The pattern of onset of failure can be clearly seen in most of the networks. For violent secret societies this transition means that the network might be initially hard to defeat, but there is a point after which efforts against it start to pay off. Because 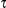 is representative of the security environment, the 9/11 network is found to be relatively ill-adapted to the more stringent security regime implemented after the attacks. Indeed, it is likely that the 9/11 attacks would have been thwarted under the current security regime since some of the nodes were captured before the attacks, but not interrogated in time to discover and apprehend the rest of the network [31]. In contrast, the cellular tree hierarchy of the FTP network is more suitable for an intermediate range of cascade risks. However, the pair-wise distances in it are too long to provide high efficiency. Therefore, its fitness is comparatively poor in the very low and very high values of 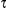.

### Designing Networks

The success of dark networks must be due to structural elements of those networks, such as cells. If identified, those elements could be used to design more resilient networks and to upgrade existing ones. Thus, by learning how dark networks organize, it will be possible to make networks such as communication systems, financial networks, and others more resilient and efficient.

**Figure 3:**
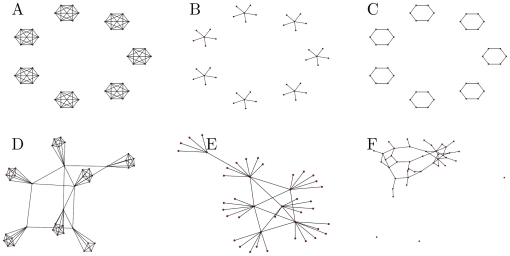
Graphs illustrating the 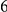 network designs. Cliques (A), Stars (B), Cycles (C), Connected Cliques (D), Connected Stars (E), and Erdos-Renyi “ER” (F). Each design is configured by just one or two parameters (the number of individuals per cell and/or the random connectivity). This enables rapid solution of the optimization problem. In computations the networks were larger (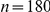 nodes).

Those identification and design problems are our next task. We propose to solve both using an approach based on discrete optimization. Let a set of graphs 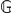 be called a “network design” if all the networks in it share a structural element. Since dark networks are often based on dense cliques, we consider a design where all the networks consist of one or multiple cliques. We consider also designs based on star-like cells, cycle-based cells and more complex patterns (see Fig. 3 and SI for the exact set of networks.)

In the first step we will find the most successful network within each design. Namely, consider an optimization problem where the decision variable is the topology 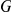 of a simple graph taken from a design 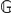. The objective is the fitness 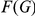:
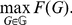
(1)In the second step we will compare the fitnesses across designs, thus identifying the topological feature with the highest fitness (e.g. star vs. clique).

This optimization problem could be used more broadly: It introduces a method for designing cascade-resilient networks for applications such as vital infrastructure networks. To apply this to a given application, one must make the design 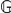 the set of all feasible networks in that domain, to the extent possible by computational constraints. In the area of terrorist networks, the model is closely related to the game-theoretic work of Lindelauf et al. [22], [32].

A complementary approach is to consider the multi-objective optimization problem in which 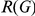 and 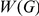 are maximized simultaneously:
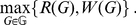
(2)The multi-objective approach cannot find the optimal network but instead produces the Pareto frontier of each design - the set of network configurations that cannot be improved without sacrificing either efficiency or resilience. The decision maker can use the frontier to make the optimal trade-off between resilience and efficiency.

## Results

### Optimal Network

**Figure 4:**
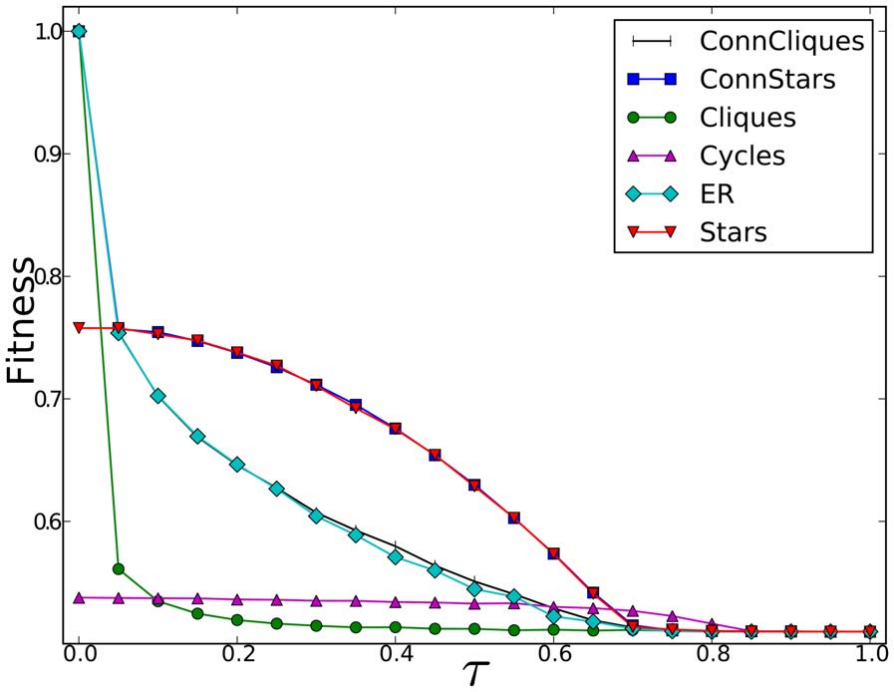
Fitness at 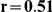 of various network designs. The Connected Stars design is the best design at all cascade risks, 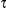. Cliques and Connected Cliques are competitive only for extreme ranges of 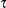. The superiority of Connected Stars over the ER (random graph) confirms the hypothesis that cells give fitness gains against cascades. The fitness of a design at each value of 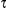 is defined as the fitness of the optimal configuration (network ensemble) within that design.

The first set of experiments compares the designs against each other under different cascade risks (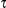), Fig. 4. At each setting of 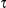, each design is optimized to its best configuration, i.e. the best cell size and connectivity, if applicable. The curves indicate the fitness of the optimal network in each design. Typically at each 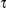 the optimal network is different from the optimal network at another 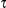. Observe that within each design, as 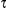 increases the fitness decreases - one cannot win when fighting cascades, only delay (see SI for proof.) In certain applications it is possible to invest in reducing the cascade propagation probability, 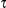. Then the curves in Fig. 4 could also be viewed as expressing the gain from efforts to reduce cascades by reducing 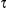 and also adapting the network structure. If the slope is steep then the gains are large.

Comparing designs to each other reveals that Connected Stars is superior to all others in fitness (Fig. 4). The design also outperforms any of the empirical networks in Fig. 2 in part because for each value of 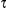 we selected the optimal network. The simpler Stars design is almost as fit, deteriorating only at extreme ranges of 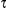. The rankings of the designs are of course dependent on the parameter values, but not strongly (see SI for proof.) Star-like designs are successful because the central node in a star acts as a cascade blocker while keeping the average distance in the star short (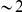). Only for sufficiently low 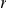, the Cliques, Connected Cliques and Connected Stars designs are superior to the Stars design. For such values of 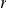 efficiency is the dominant contributor to fitness. High weighting for efficiency benefits the former designs where efficiency can be 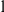 by constructing a fully connected (complete) graph (see SI for analytic results.) In the star design efficiency is lower, reaching 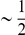 (when all nodes are placed in a single large star).

It has been long conjectured that cells provide dark networks with high resilience. Indeed, this is probably the reason why we found that dark networks have higher fitnesses than other networks. But cells also reduce the efficiency of a network since they isolate nodes from each other. To rigorously determine the net effect of cells, we compare the ER design (random graphs) to the Connected Stars design. ER is a strict subset of Connected Stars but only Connected Stars has cells. Therefore it is notable that Connected Stars has a higher fitness than ER, often significantly so. Indeed, cells must be the cause of higher fitness because cells are the only feature in Connected Stars that ER lacks.

### Properties of Optimal Networks

**Figure 5:**
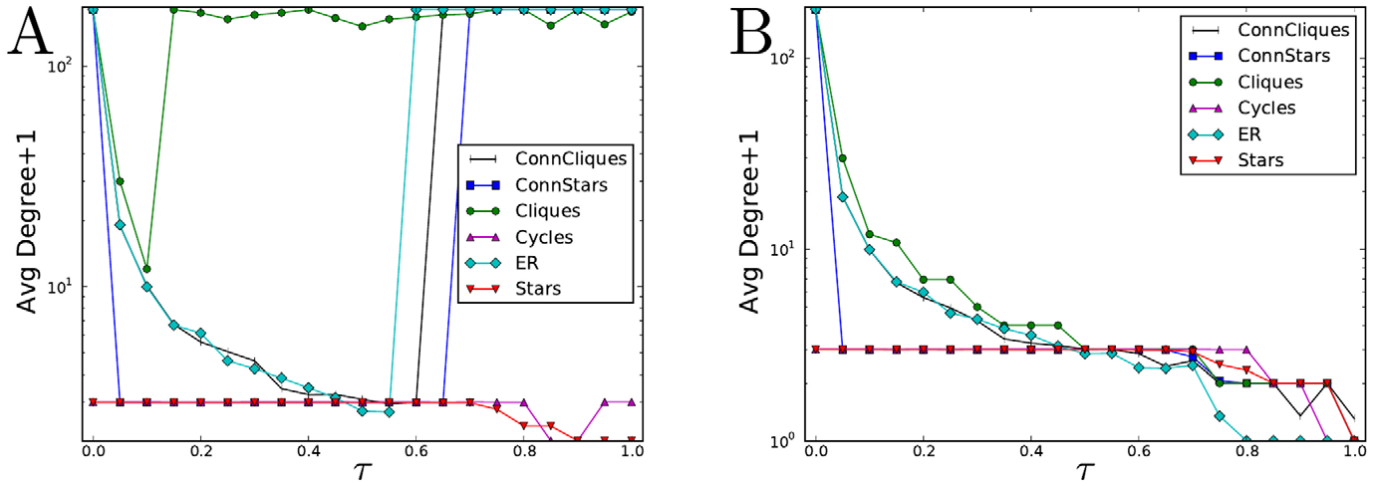
Average degree in the optimal configuration of each design. At 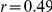 (A) the optimization prefers networks that have high efficiency while at 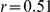 (B) the preference is for resilience. In (B) the average degree diminishes monotonically to compensate for increasing cascade risk. In (A) most designs have a threshold 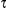 at which they jump back to a completely-connected graph because structural cascade resilience becomes too expensive in terms of efficiency.

Many properties of the optimal networks such as resilience, efficiency and edge density show rapid phase transitions as 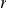 is changed. For example, in the Cliques design when 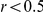 the optimal network has high density that maximizes efficiency, whereas for 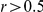 it is sparse and maximizes resilience (Fig. 5).

Intuition may suggest that the networks grow more sparse as cascade risk grows. Instead, the trend was non-monotonic (Fig. 5). For 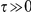 and 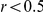 Cliques, Connected Cliques and Connected Stars became denser, instead of sparser, and for them the most sparse networks were formed in the intermediate values of 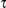 where the optimal networks achieve both relatively high resilience and high efficiency. At higher 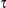 values, when 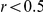 it pays to sacrifice resilience because fitness is increased when efficiency is made larger through an equal or lesser sacrifice in resilience. The Stars design does not show a transition at 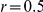 because it is hard to increase efficiency with this design.

### Multi-objective Optimization

**Figure 6:**
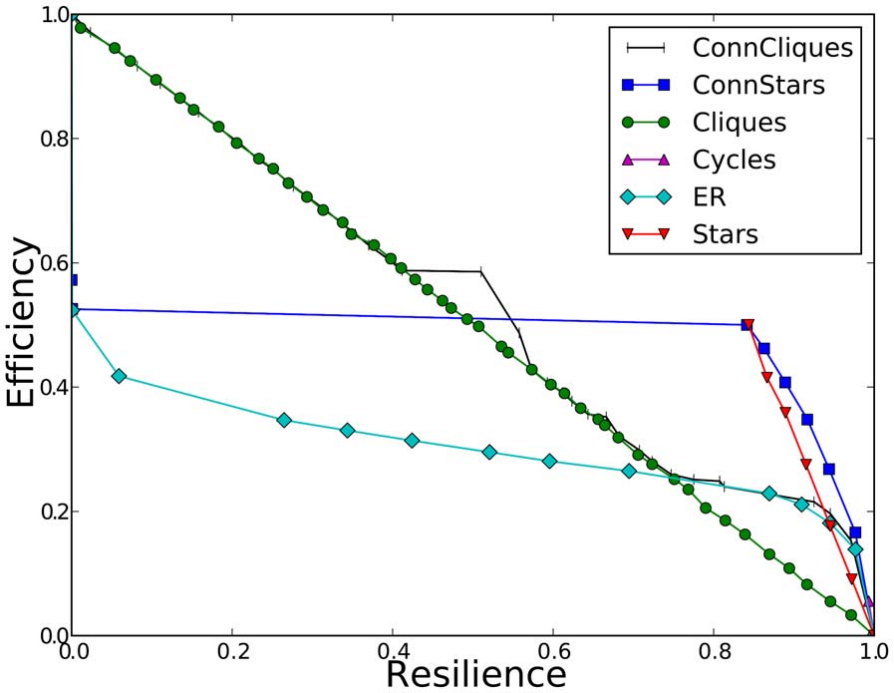
The Pareto frontiers of various network designs (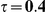). The configurations of the Connected Stars design dominate over other designs when the network must achieve high resilience. However, designs based on cliques are dominant when high efficiency is required. Several designs show sharp transitions where at a small sacrifice of efficiency it is possible to achieve large increases in cascade resilience.

A complementary perspective on each design is found from its Pareto frontier of resilience and efficiency (Fig. 6). Typically a design is dominant in a part of the Resilience-Efficiency plane but not all of it. The Stars and Connected Stars designs can access most of the high resilience-low efficiency region. In contrast, the Cliques and Connected Cliques can make networks in the medium resilience-high efficiency regions.

The sharp phase transitions discussed earlier are seen clearly: along most of the frontiers, if we trace a point while decreasing resilience, there is a threshold at which a small sacrifice in resilience gives a major gain of efficiency. More generally, consider the points where the frontier is smooth. By taking two nearby networks on the frontier one can define a rate of change of efficiency with respect to resilience: 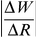. The ratio can be used to optimize the network without using the parameter 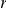. When 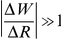 the network optimizer should choose to reduce to the resilience of the network in order to achieve great gains in efficiency; when 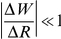 efficiency should be sacrificed to improve resilience.

## Discussion

The analysis above considered both empirical networks and synthetic ones. The latter were constructed to achieve structural cascade resilience and efficiency. In contrast, in many empirical networks the structure emerges through an unplanned growth process or results from optimization to factors such as cost rather than blocking cascades. Without exception the synthetic networks showed higher fitness values despite the fact that they were based on very simple designs. This suggests that network optimization can significantly improve the fitness and cascade resilience of networks. It means that such an optimization process can indeed be an effective method for designing a variety of networks and for protecting existing networks from cascades.

Many empirical networks also have power-law degree distributions [26]. Unfortunately, this feature significantly diminishes their cascade resilience: the resulting high-degree hubs make the networks extremely vulnerable to cascades once 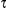 is slightly larger than 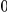 [1], [2].

In some successful synthetic networks the density of edges increased when the cascade risk 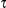 was high. This phenomenon has interesting parallels in non-violent social movements which are often organized openly rather than as secret underground cells even under conditions of severe state repression [33]. This openness greatly facilitates recruitment and advocacy, justifying the additional risk to the participants, just like the sacrifice of resilience to gain higher efficiency is justified under 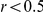 conditions.

There are other important applications of this work, such as the design of power distribution systems. For power networks, the definition of resilience and efficiency will need to be changed. It would also be necessary to use much broader designs and optimization under design constraints such as cost. Furthermore, this work could also be adapted to domains of increasing concern such as financial credit networks, whose structure may make them vulnerable to bankruptcies [14], [15].

## Methods

### Measuring Resilience

Research on graph theory has led to the development of a variety of metrics of robustness or resilience [34] but here unlike in many other studies the interest is in resilience to cascades and not to disconnection. One particularly important and well-characterized class of cascades are those that start at a single node and then spread probabilistically to neighboring nodes possibly reaching a large fraction of the network, termed the SIR model and percolation [26]. Under this model, resilience can be defined based on the expected size of the surviving network:
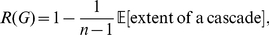
(3)where “extent of a cascade” refers to the ultimate number of new cases created by a single failed node. For simplicity, cascades are assumed to start at all nodes with uniform probability.

### Measuring Efficiency

For many applications the distance between pairs of nodes in the network is one of the most important determinants of the network's efficiency (see e.g. [32], [35], [36].) When nodes are separated by short distances they can easily communicate and distribute resources to each other. This idea motivates the following “distance-attenuated reach” metric. For all pairs of nodes 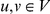, weigh each pair by the inverse of its internal distance (the number of edges in the shortest path from 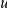 to 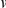) taken to power 
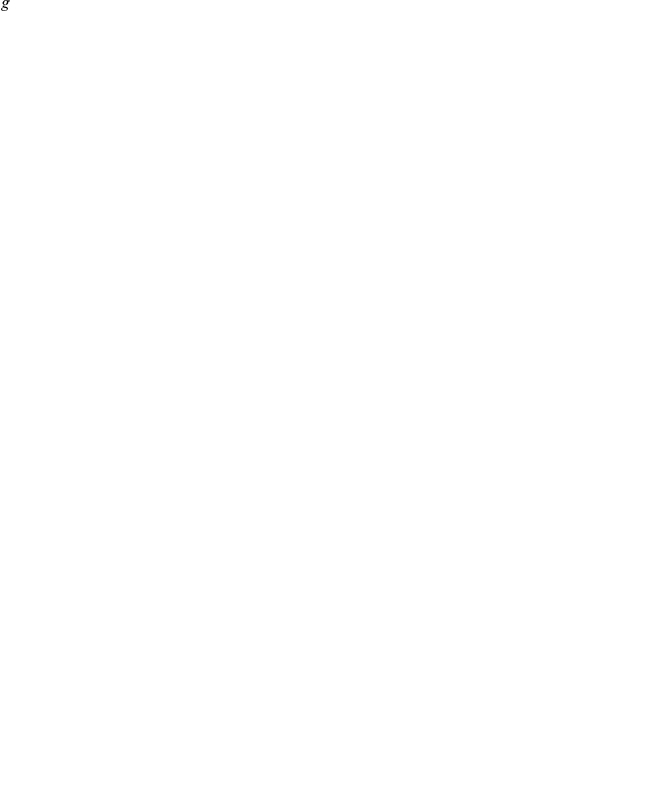
:
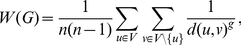
(4)Normalization by 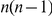 ensures that 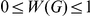, and only the complete graph achieves 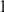. As usual, for any node 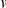 with no path to 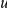, set 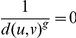. The parameter 
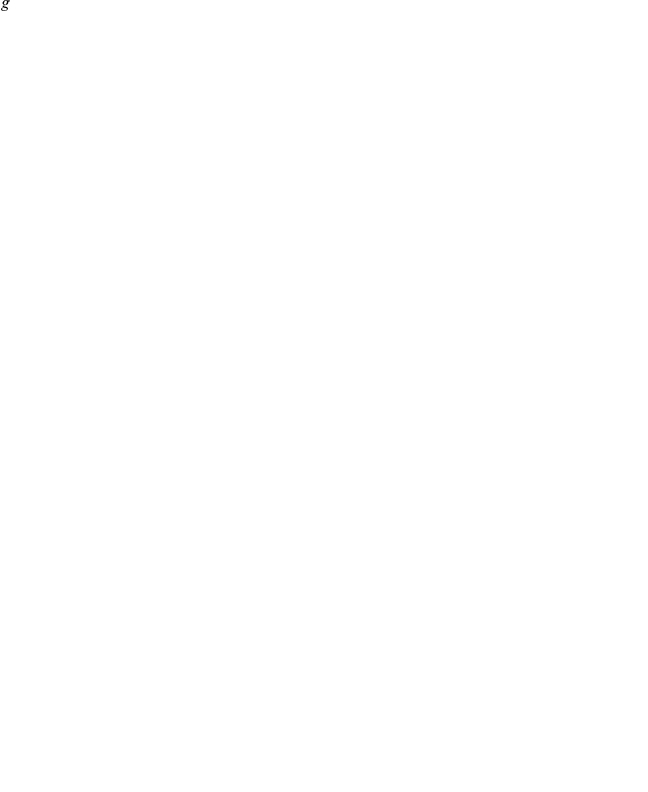
, “connectivity attenuation” represents the rate at which distance decreases the connectivity between nodes. In the experiments above 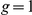.

An appendix (File S1) is linked to this article. It contains detailed information about the optimization methodology, the simulation process, and sensitivity as well as rigorous justification of quantitative claims.

## Supporting Information

File S1 Optimizing topological cascade resilience based on the structure of terrorist networks.
(1.11 MB PDF)
