# Supplementary material for: Optimizing Topological Cascade Resilience Based on the Structure of Terrorist Networks
Source: PLoS One. 2010 Nov 10;5(11):e13448. doi: 10.1371/journal.pone.0013448 (PMC2978083; doi:10.1371/journal.pone.0013448)
Supplement: File S1 — Optimizing topological cascade resilience based on the structure of terrorist networks. (1.11 MB PDF) [file pone.0013448.s001.pdf]

Optimizing topological cascade resilience based on the structure of terrorist networks - Supporting Information File S1

Alexander Gutfraind

S1 Resilience and Efficiency of Empirical Networks

It is interesting to compare the empirical networks to each other in their efficiency and resilience (Fig. S1). Note that FTP and 9/11 networks are not the most resilient, but they strike a good balance between resilience and efficiency. The advantages of the two networks over other networks are not marginal, implying that their advantages in fitness are not sensitive to the choice of r. Of course, they are optimized for particular combinations of r and τ, and will no longer be very successful outside that range. For instance, in the range of high r and high τ networks with multiple connected components would have higher fitness because they are able to isolate cascades in one component.

S2 Resilience and Efficiency of Weighted Networks

In some networks, each edge (u, v) carries a distance weight $D_{uv} > 0$. The smaller the distance, the closer the connection between u and v. To compute the fitness of those networks, we now introduce generalizations of resilience and

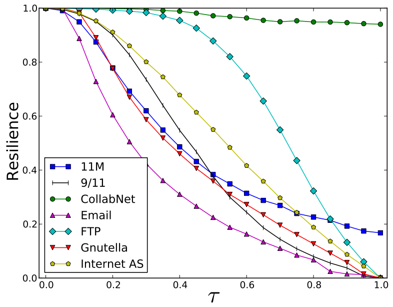

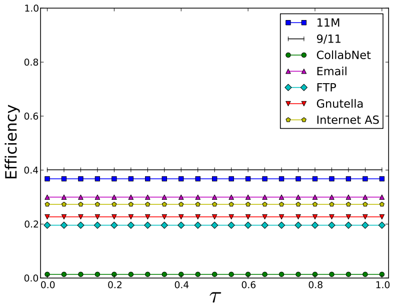

Fig. S1. Resilience and Efficiency of the real networks. The fittest networks are not always the most resilient.

efficiency. Those reduce to the original definitions for unweighted networks when $D_{uv} = 1$, while capturing the effects of weights in the weighted networks.

The original definition of resilience was built on a percolation model where the failure of any node leads to the failure of its neighbor with probability τ. In the weighted network more distant nodes should be less likely to spread the cascade. Thus we make the probability of cascade through (u, v) to be $\min(\tau/D_{uv}, 1)$.

The efficiency was originally defined as the sum of all-pairs inverse geodesic distances, normalized by the efficiency of the complete graph. In the weighted network, both the distance and the normalization must be generalized. To compute the distance $d(u, v)$ we consider the weights on the edges D and apply Dijkstra's algorithm to find the shortest path. Normalization too must consider D because a weighted graph with sufficiently small distances could outperform the complete graph (if all the edges of the latter have $D_{ij} = 1$). Therefore, we weigh the efficiency by the harmonic mean H of the edges (E) of the graph:

$$W(G) = \frac{H(G)}{n(n-1)} \sum_{u \in V} \sum_{v \in V \setminus \{u\}} \frac{1}{d(u,v)^g}, \tag{S1}$$

where

$$H(G) = \frac{|E|}{\sum_{(u,v)\in E} \left(\frac{1}{D_{uv}}\right)^g} .$$

The harmonic mean ensures that for any D, the complete graph has $W(G) = 1$.

Having defined generalized resilience and efficiency we can evaluate the standard approach to dark network, which represents them as binary graphs $D_{uv} \in \{0, 1\}$, rather than as weighted graphs. The former approach is often taken because the information about dark networks is limited and insufficient to estimate edge weights.

Fortunately, in two cases, the 9/11 network and the 11M network [1, 2] the weights could be estimated. The 9/11 data labels nodes as either facilitators or hijackers. Hijackers must train together and thus should tend to have a closer relationship. Thus set $D_{uv} = 2, 1, 0.5$ if the pair u, v includes zero, one or two hijackers, respectively. The 11M network is already weighted ($Z_{uv} = 1, 2, 3 \dots$) based on the number of functions each contact (u, v) serves (friendship, kin, joint training etc). We mapped those weights to D by $D_{uv} = 2/Z_{uv}$. In both networks the transformation was so that the weakest ties have weight 2, giving them greater distance than in the binary network, while the strongest ties are shorter than in the binary network.

Figure S2 compares the fitnesses, resiliences and efficiencies of the weighted and binary representations. It shows that for both networks, the fitnesses of the binary representation lies within 0.15 of the fitness of the weighted representation and for some τ much closer. The efficiency measures are even more close (within 0.05). The behavior of resilience is intriguing: for the 9/11 network the weighted representation shows more gradual decline as a function of cascade risk when compared to the binary representation. For the 11M network the decline

is actually slightly more sharp in the weighted representation. Structurally, the 11M network has a center (measured by betweenness centrality) of tightly knit-nodes (very short distances), while the 9/11 network is more sparse at its center, increasing its cascade resilience. This effect explains the direction of the error in the binary representation. Based on those two examples, it appears that the binary representation does not have a systematic bias, and may even underestimate the fitness of dark networks.

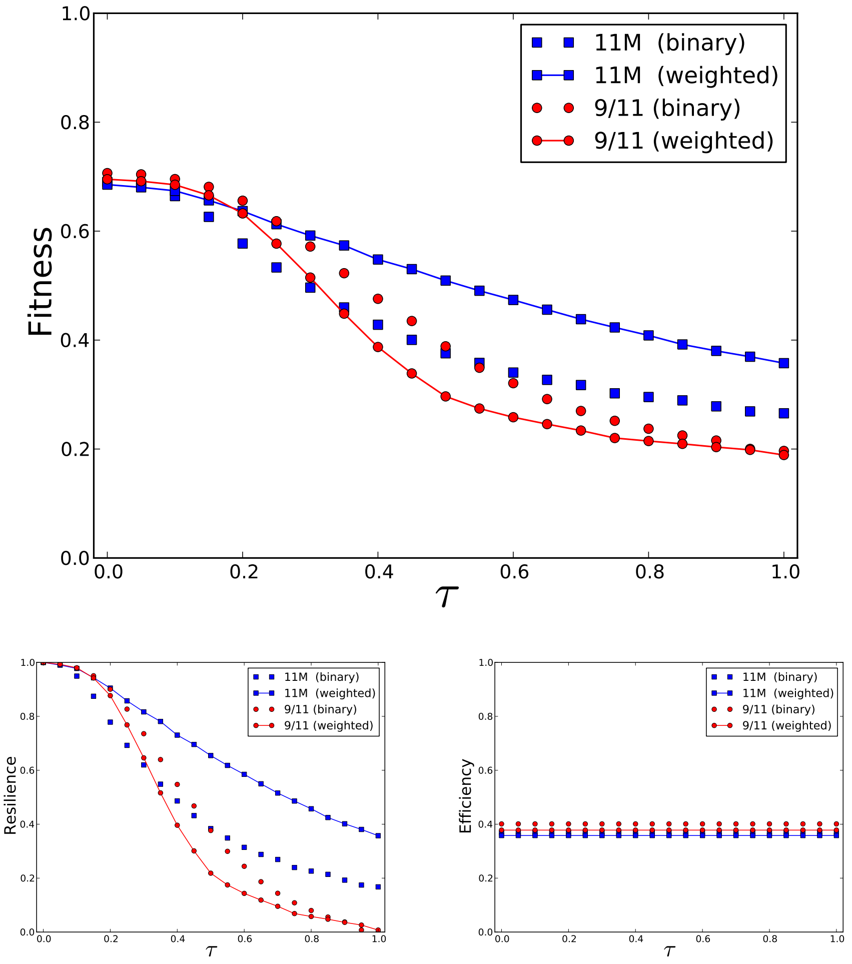

Fig. S2. Fitness, Resilience and Efficiency of two dark networks ($r = 0.51$), comparing binary and weighted representations. The binary representation matches the weighted representation within 0.15, and typically closer.

S3 Network Designs

We considered networks on $n = 180$ nodes constructed through 6 simple designs, chosen both based on empirical findings (see e.g.[3, 4]) as well as the possibility of analytic tractability in some cases. When more data becomes available on dark networks, it will become possible to extract additional subgraphs with statistical validity.

Three of the designs are based on identical "cells": each cell is either (a) a clique (a complete graph), (b) a star (with a central node called "leader"), and (c) a cycle (nodes connected in a ring). Each of these have a single parameter, k - the number of nodes in the cell. Recent research suggests that under certain assumptions constructing networks from identical cells is optimal [5]. Let us also consider n-node graphs consisting of (d) randomly-connected cliques (sometimes termed "cavemen"), and (e) randomly-connected stars, in both cases according to probability p . Consider also (f) the simpler and well-studied Erdos-Renyi (ER) random graph with probability p (see figure in main text). By considering different structures for the cells we determine which of those structures provides the best performance.

The above palette of designs is sufficient for the current study's purpose of introducing an approach which could be applied to different domains, as well as begin constructing a theory to address cascade resilience. There are surely applications where if the objective is to design cascade-resilient networks, one ought to both reject some of the designs above for application-specific reasons and also to introduce additional designs. The task of taking other designs can be done by merely changing the computer program for generating the networks. Fruitful future research in cascade-resilient network is to attack the most general problem of finding the best network on n nodes. Unfortunately, this search space is exponential and probably non-convex.

Research on social networks indicates that resilience and efficiency might be just two of several design criteria that also include e.g. "information-processing requirements", that impose additional constraints on network designs [6]. In the original context "information-processing" refers to the need to have ties between individuals involved in a particular task, when the task has high complexity. Each individual might have a unique set of expertise into which all the other agents must tap directly. Generalizing from sociology, such "functional constraints" might considerably limit the flexibility in constructing resilient and efficient networks. Such functional constraints could be addressed by looking at a palette of network designs which already incorporate such constraints or using innovative techniques from convex optimization [7].

The solution to the optimization problem is found by setting each of the parameters k (and when possible p) to various values. Each design D has "configurations" $C_1^D, C_2^D, \ldots$ each specifying the values of the parameters. Each configuration C_i^D is inputted to a program that generates an ensemble of $1 - 10$ networks, whose average performance provides an estimate of the fitness of C_i^D. The number of networks was 10 for networks with parameter p because there is higher variability between instances. The coefficient of variation (CV) in the

fitness of the sample networks was monitored to ensure that the average is a reliable measure of performance. Typically CV was < 0.2 except near phase transitions of connectivity and percolation.

Optimization was performed using grid search. Alternative methods (e.g. Nelder-Mead) were considered but grid search was chosen despite its computational cost because it suffers no convergence problems even in the presence of noise (present due to variations in topology and contagion extent), and collects data useful for sensitivity analysis and multi-objective optimization. The sampling grid was as follows. In designs consisting of cells of size k, cell size was set to all integer values in $[1, 180]$. If k did not divide 180, a cell of size $< k$ was added to ensure that the number of nodes in the graph is 180. The number of nodes is 180 because 180 is a highly-composite number and so it offers many networks of equally-sized cells. In general, normalization by n in the definitions of resilience and efficiency ensures that even when the number of nodes is tripled the effect of network size on fitness is very small for the above designs (around ± 0.05 in numerical experiments). In designs containing a parameter of connectivity p, it was set to all multiples of 0.05 in $[0, 1]$, with some extra points added to better sample phase transitions. The grid search algorithm results are readily used to compute the Pareto frontier using the ϵ-balls method [8] ($\epsilon = 0.01$).

The resilience metric is most easily computed by simulation where a node is selected at random to be "infected", and the simulation is run until all nodes are in states S or R, and none is in state I. In the simplest version of the SIR cascade model, which we adopt, each node in the graph can be in one of three states "susceptible", "infected" and "removed" designated S, I and R respectively (these names are borrowed from epidemiology). Time is described in uniform discrete steps. A node in S state at time t stays in this state, unless a neighbor "infects" the node, causing it to move to state I at time $t + 1$. Specifically, a node in state S at time t has probability τ of turning to I state at time $t + 1$ for each adjacent node in state I at time t. Finally, a node in I state at time t always becomes R at time $t + 1$. Once in state R, the node remains there for all future times. It is possible to consider an alternate model where the rate of transition $I \to R$ takes more than one time step, but adding this effect would mostly serve to increase the probability of transmission, which is already parametrized by τ [9, 10].

A cascade/contagion that starts at a single node would run for up to n steps, but usually much fewer since typically $\tau < 1$ and/or the graph is not connected. To achieve good estimate of the average extent, the procedure was replicated 40 times, and then continued as long as necessary to achieve an error of under ± 0.5node with a 95% confidence interval [11].

An analytic computation of the cascade extent metric was investigated. It is possible in theory because the contagion is a Markov process with states in the superset of the set of nodes, 3^n. Unfortunately, such a state space is impractically large. When G is a tree, then an analytic expression exists[1], and it might be feasible when the treewidth is small [12, 9]. However, for many

[1] Specifically, the mean contagion size is $1 + \frac{pG_0'(1)}{1-pG_1'(1)}$, where $G_0(x)$ generates the degree

graph designs the tree approximation is not suitable. Another possible approach is to represent the contagion approximately as a system of differential equations which can be integrated numerically [13] . These possibilities were not pursued since the simulation approach could be applied to all graphs, while the errors of the analytic approaches are possibly quite large.

S4 Continuity of Fitness in r

We now justify the claim that fitness is continuous in r. In fact, we will prove the stronger property of Lipschitz-continuity. Notice that the claim is not about the continuity of fitness of a single configuration as a function of r but rather that:

Claim: $f(r) = \max_{G \in \mathbb{G}} F(G, r)$ is Lipschitz-continuous for $r \in [0, 1]$.

Proof: The argument constructs a bound on the change in f in terms of the change in r. Consider an optimal configuration C_1 of a design for $r = r_1$ and let its fitness be $f_1 = F(C_1, r_1)$ (there is slight abuse of notation since C is a configuration, whose fitness is the average fitness of an ensemble of graphs).
Observation 1: consider the fitness of C_1 at $r = r_2$. Because C_1 is fixed and the metrics are bounded ($0 \leq R \leq 1$ and $0 \leq W \leq 1$), the fitness change is bounded by the change in r:

$$\begin{aligned} |f_1 - F(C_1, r_2)| &= |r_1 R(C_1) + (1 - r_1) W(C_1) \\ &\quad -r_2 R(C_1) - (1 - r_2) W(C_1)| \\ &= |(r_1 - r_2) R(C_1) - (r_1 - r_2) W(C_1)| \\ &\leq |r_1 - r_2| \; . \end{aligned}$$

Observation 2: let C_2 be the optimal configuration for $r = r_2$ and let $f_2 = F(C_2, r_2)$. Since C_2 is optimal for $r = r_2$ it satisfies: $f_2 \geq f(C_1, r_2)$, and so $-f_2 \leq -F(C_1, r_2)$. It follows that $f_1 - f_2 \leq f_1 - F(C_1, r_2)$. Take the absolute value of the right hand side and apply Observation 1 to get the bound: $f_1 - f_2 \leq |r_1 - r_2|$.
Observation 3: applying the argument of Observations 1&2 but reversing the roles of C_1 and C_2 implies that $f_2 - f_1 \leq |r_1 - r_2|$.

Observations 2&3 give $|f_1 - f_2| \leq |r_1 - r_2|$, proving the result.

S5 Additional Performance Results

A plot of the fitness of various designs is in Fig. S3. Notice that for $r = 0.25$ Cliques outperform Stars. The resilience and efficiency are in Fig. S4 and S5.

---

distribution and $G_1(x) = \frac{G_0'(x)}{G_0'(1)}$ generates the probability of arrival to a node [9].

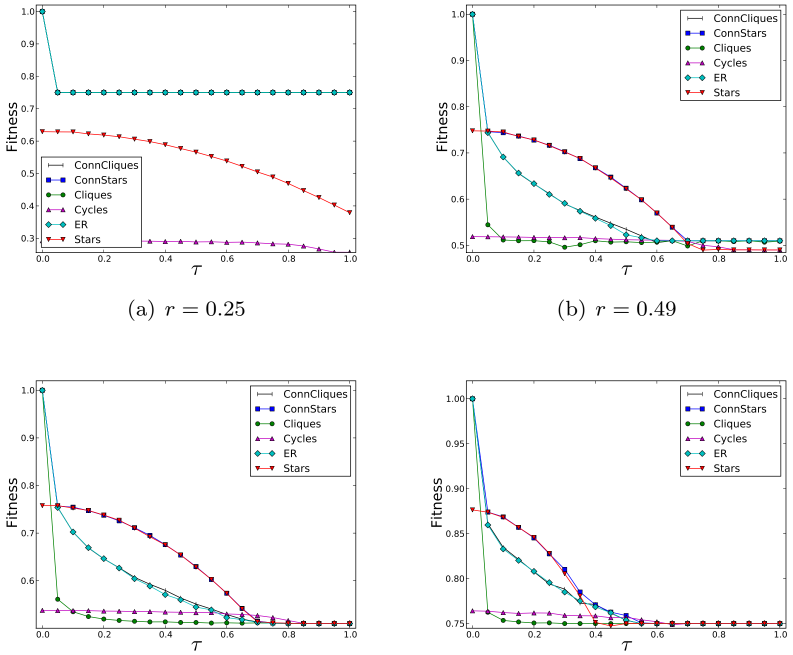

(a) $r = 0.25$ (b) $r = 0.49$

(c) $r = 0.51$ (d) $r = 0.75$

Fig. S3. Fitness of the optimal configuration. For very low r Cliques outperform Stars.

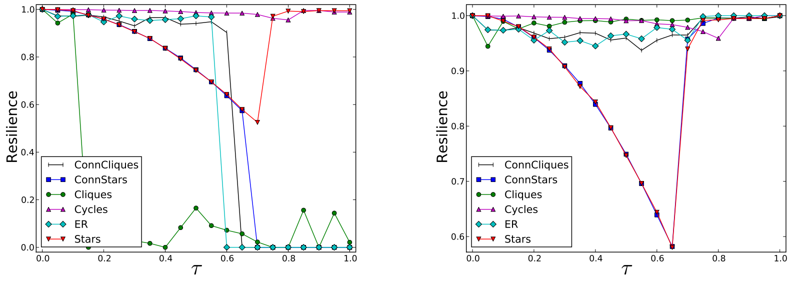

(a) $r = 0.49$ (b) $r = 0.51$

Fig. S4. Resilience of the optimal design.

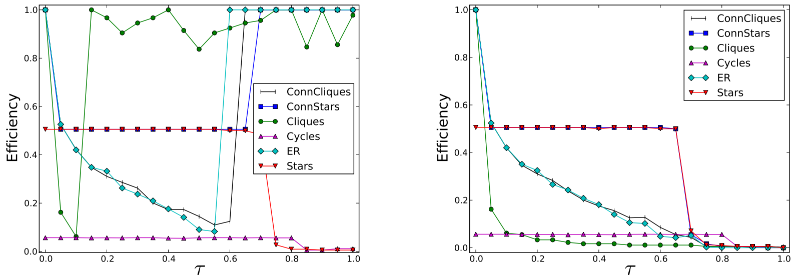

(a) $r = 0.49$ (b) $r = 0.51$

Fig. S5. Efficiency of the optimal design.

The success of the Stars design could be analyzed more qualitatively. The fitness function combines resilience $R(G)$ which decreases when the graph becomes more strongly connected, and efficiency $W(G)$ which decreases when the graph becomes more sparse. The optimality of the star-based designs is due to a good trade-off between R and W: the central node in each cell (its "leader") provides a good firewall against cascades because in each cell most pairs are separated by distance of 2, but this separation reduces efficiency only modestly. In the Cliques design the separation is 1 (too short for resilience), and in the Cycles design it is too long ($\sim$a quarter of cell size k).

Mathematically, the existence of a non-trivial solution is due to the different functional relationships. To first-order approximation, efficiency decreases inversely with average distance,$\Theta\left(\frac{1}{\text{avg distance}^g}\right)$ while cascade extent decreases exponentially, $\Theta\left(\tau^{\text{avg distance}}\right)$, for $\tau < 1$ assuming a bounded number of alternative paths). For example, for the star design $R = O(1 - \tau^2)$ and $W = O(2^{-g})$ as $n = k \to \infty$. Therefore, the optimal network's structure exploits the exponential decrease in cascades without sacrificing too much efficiency. In the range $\tau \in [0.2, 0.7]$ and $r \approx 0.5$, an average distance of ≈ 2, as in the star graph, might be optimal (cf. [14]).

S6 Monotonicity of Fitness

Proposition: Let $f(\tau)$ be the highest attainable fitness within a fixed network design $\mathbb{G}$, for cascade probability τ:

$$f(\tau) = \max_{G \in \mathbb{G}} \left[\underbrace{rR(G,\tau) + (1-r)W(G)}_{F(G,\tau)} \right]$$

Then $f(\tau)$ is a non-increasing function of τ.

Proof of Proposition: The proof relies on the simple claim that resilience of networks does not increase when τ increases [15]. The claim is equivalent to the result that for a given graph G increasing τ does not decrease the expected extent of cascades. The remainder is almost trivial: we need to show that when the fitness of all the points on the space (all graphs) has been made smaller or kept the same (by increasing τ), the new maximum value would not be greater than in the old space. Rigorously, assume by contradiction that $\tau_+ > \tau$ and fitness *increased*, namely*:*

$$f(\tau_+) > f(\tau)\,. \tag{S2}$$

Let $G^*_{\tau_+}, G^*_\tau$ by any two optimal networks for τ_+ and τ, respectively, namely: $G^*_{\tau_+} \in \text{argmax}_{G\in\mathbb{G}}\left[rR(G,\tau_+) + (1-r)W(G)\right]$ and $G^*_\tau \in \text{argmax}_{G\in\mathbb{G}}\left[rR(G,\tau) + (1-r)W(G)\right]$. By optimality of G^*_τ at τ get that it must be at least as good at τ as $G^*_{\tau_+}$:

$$\underbrace{F(G^*_\tau,\tau) - F(G^*_{\tau_+},\tau)}_{\equiv\Delta} \geq 0\,. \tag{S3}$$

The claim implies that:

$$R(G^*_{\tau_+},\tau) \geq R(G^*_{\tau_+},\tau_+)\,. \tag{S4}$$

Expanding Δ:

$$\begin{aligned}
\Delta &= F(G^*_\tau,\tau) - \left[rR(G^*_{\tau_+},\tau) + (1-r)W(G^*_{\tau_+})\right] \\
&\leq F(G^*_\tau,\tau) - rR(G^*_{\tau_+},\tau_+) - (1-r)W(G^*_{\tau_+}) \text{ by } (S4) \\
&= F(G^*_\tau,\tau) - F(G^*_{\tau_+},\tau_+) \\
&< 0 \text{ by the hypothesis (S2).}
\end{aligned}$$

This contradicts Ineq. S3. The argument is easy to generalize. One could apply this method to the parameter g of attenuation, showing that fitness is non-increasing when attenuation is increased.

S7 Analytic Results

It is easy to analytically derive the values of the resilience, efficiency (and hence fitness) functions for certain simple designs: the Cycles and the Stars designs. These are useful to gain deeper insight into the effect of parameters. Recall that n is the number of nodes and k is the number of nodes per cell. For $k = 1$, in both designs $R = 1$ and $W = 0$. When $k \geq 2$, it is easy to verify that for the Cycle design:

$$\begin{aligned}
R(n,k,\tau) &= 1 - \frac{1}{n-1}\left[2\tau\frac{1-\tau^{k-1}}{1-\tau} - (k-1)\tau^k\right] \\
W(n,k,g) &= \frac{1}{n-1}\begin{cases} \frac{1}{(\frac{k}{2})^g} + 2\sum_{j=1}^{\frac{k}{2}-1}\frac{1}{j^g} & \text{k even} \\ 2\sum_{j=1}^{\frac{k-1}{2}}\frac{1}{j^g} & \text{k odd}\end{cases}
\end{aligned}$$

and for the Stars design:

$$
\begin{aligned}
R(n,k,\tau) &= 1 - \frac{1-\frac{1}{k}}{n-1}\left[2+\tau(k-2)\right]\tau \\
W(n,k,g) &= \frac{1-\frac{1}{k}}{n-1}\left[2+2^{-g}(k-2)\right] .
\end{aligned}
$$

These expressions are not readily useful for continuous optimization since k is discrete, but they can be used to identify phase transitions. Thus, they help inform optimization for designs where no analytic expression is available. The ER and Cliques designs are also analytically tractable (see e.g. [16] for expected cascade extent).

In the Stars design, when R and W are weighted equally ($r = \frac{1}{2}$), fitness takes a relatively simple form: $F = \frac{1}{2} + \frac{1}{2}\frac{1-\frac{1}{k}}{n-1}\left[2(1-\tau) + (2^{-g} - \tau^2)(k-2)\right]$. This implies that increasing cell size k, for k large, improves fitness iff $2^{-g} - \tau^2 > 0$. Hence the optimal configuration has one cell ($k = n$), until a threshold near $\tau = 2^{-g/2}$ (for $g = 1$, approximately 0.71). This agrees with the findings in Fig. S6. Also, the rate of change in fitness with respect to τ, $\frac{dF}{d\tau} = \frac{1-\frac{1}{k}}{n-1}\left[-2 - 2\tau(k-2)\right]$, is always negative, as expected on more general grounds (see sec. S6). It is linear in τ (because it is a tree graph) but superlinear in k (because of the mutual hazard induced by adding nodes to cells).

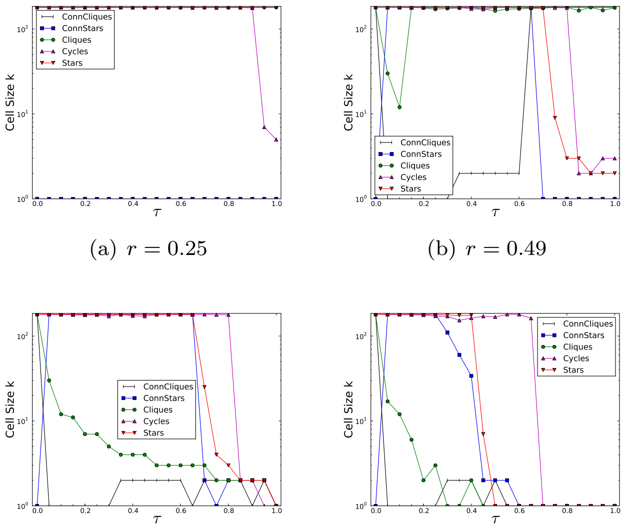

(a) $r = 0.25$ (b) $r = 0.49$

(c) $r = 0.51$ (d) $r = 0.75$

Fig. S6. Cell size k in the optimal configuration of each design. Cell size makes many sharp transitions.

S8 Configuration of the Optimal Design

As τ is varied, the optimal configuration changes. This section shows the resulting changes in the values of the parameters k (cell size) and p (connectivity). In other words, it indicates how each of the designs ought to be configured to attain optimal fitness, as a function of resilience weighting, r, and cascade probability, τ.

The cell size parameter k is non-monotonic for various designs when $r = 0.49$ (Fig. S6). For example, for the Connected Cliques design, at low contagion risk ($\tau < 0.1$), k is high (comparable to the size of the network, i.e. $k \to n$), then it falls to a small number. At high contagion risk ($\tau > 0.6$) the network is again highly connected again with $k \to n$. Thus for $\tau \to 1$, the optimal network is the fully-connected graph.

In general, designs involving both the p and k parameters show an interplay between the two (Fig. S7). For example, in the Connected Stars design under $r < 0.5$ there are two phase-transitions in connectivity p: as τ increases at $\tau \to \tau_l^* \approx 0.1$ it transitions from a connected graph to disconnected cells, and at $\tau \to \tau_u^* \approx 0.7$ back to full connectivity. If $r > 0.5$ the second transition is extinguished. The data requires care to interpret. For example, in the Connected Stars design $\tau \in [0.1, 0.65]$, when $r > 0.5$ the fluctuations in the p are noise because there is a single cell and a single cell leader ($k = n$), and so the parameter p has no effect. For sensitivity analysis see section S9.

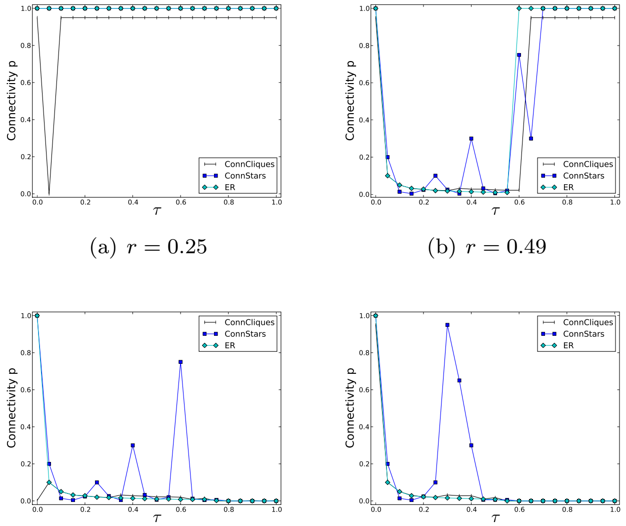

(a) $r = 0.25$ (b) $r = 0.49$

(c) $r = 0.51$ (d) $r = 0.75$

Fig. S7. Connectivity p in the optimal configuration of each design. Non-monotonic trends reflect structural transitions and sometimes also interplay with cell size.

S9 Sensitivity Analysis

It is desirable to determine how much variability exists within the optimal parameter values. Only if the variability is low the optimal configuration provides valuable information for network design. As proxy of robustness of the estimates, consider the space of configurations whose fitness ≥ 0.95 of the fitness of the optimal solution. The robustness of a parameter is measured by the parameter's standard deviation within this space (the unbiased estimate).

The results are in Figs. S8,S9,S11,S10. Overall, as one would expect, the properties are more variable near the transition point $r = 0.5$, as compared to r values away from $r = 0.5$. Moreover, variability is high within each design whenever the design undergoes a phase transition, since multiple different phases have nearly equal fitness. Another source of variance is found in designs with two parameters. These are more variable than designs with a single parameter because the former can reproduce some of the same graphs with many different parameter settings - the parameters have non-orthogonal effects. Finally, designs with low fitness for all configurations (like Cycles) show high variability since all configurations show uniform low performance.

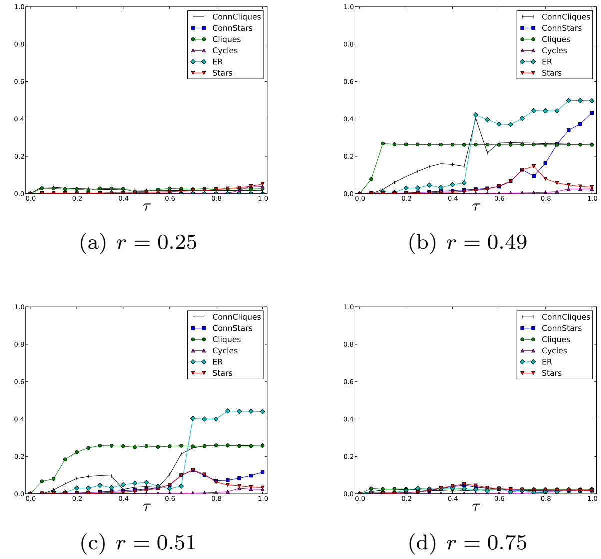

(a) $r = 0.25$ (b) $r = 0.49$

(c) $r = 0.51$ (d) $r = 0.75$

Fig. S8. Standard deviation in resilience within the top 5% of solutions.

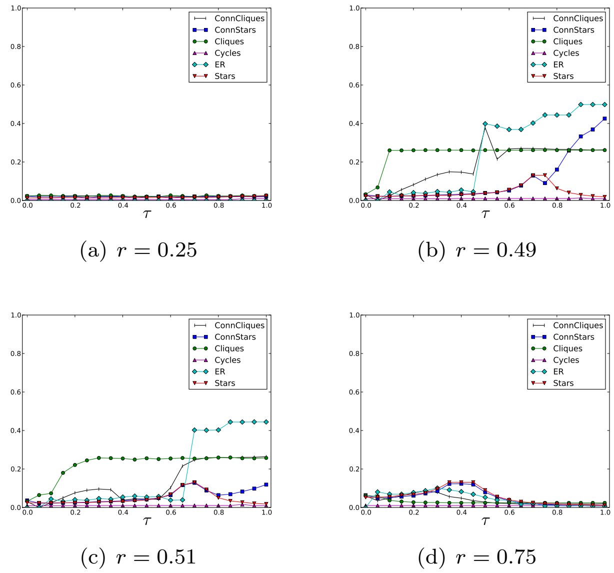

(a) $r = 0.25$ (b) $r = 0.49$

(c) $r = 0.51$ (d) $r = 0.75$

Fig. S9. Standard deviation in efficiency within the top 5% of solutions.

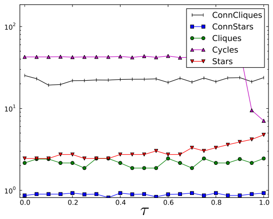

(a) $r = 0.25$

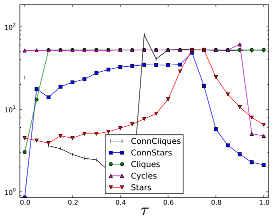

(b) $r = 0.49$

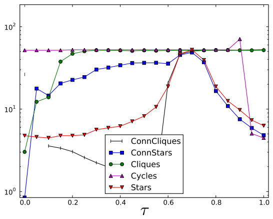

(c) $r = 0.51$

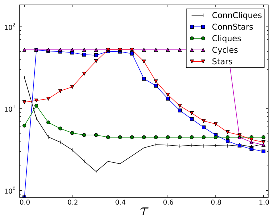

(d) $r = 0.75$

Fig. S10. Standard deviation in cell size k of the top 5% of solutions.

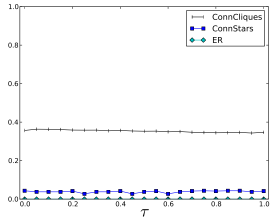

(a) $r = 0.25$

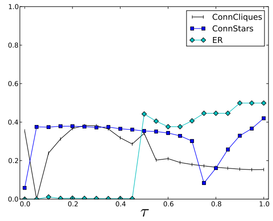

(b) $r = 0.49$

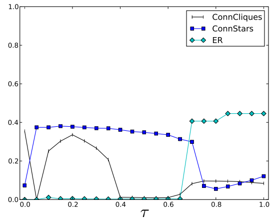

(c) $r = 0.51$

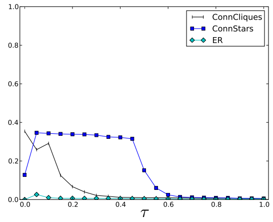

(d) $r = 0.75$

Fig. S11. Standard deviation in connectivity p of the top 5% of solutions.

References

[1] Krebs VE (2002) Mapping networks of terrorist cells. Connections 24: 43–52.

[2] Rodriguez J (2004). The march 11th terrorist network: In its weakness lies its strength. Working Papers EPP-LEA, University of Barcelona.

[3] Arquilla J, Ronfeld D (2001) Networks and Netwars: The Future of Terror, Crime, and Militancy. Santa Monica, CA: RAND Corporation.

[4] Carley KM (2006) Destabilization of covert networks. Comput Math Organiz Theor 12: 51–66.

[5] Goyal S, Vigier A (2010) Robust networks. URL `http://sticerd.lse.ac.uk/seminarpapers/et11032010.pdf`. Working paper http://sticerd.lse.ac.uk/seminarpapers/et11032010.pdf.

[6] Baker WE, Faulkner RR (1993) The social organization of conspiracy: Illegal networks in the heavy electrical equipment industry. American Sociological Review 58: 837–860.

[7] Johnson JK, Chertkov M (2010). A majorization-minimization approach to design of power transmission networks. Submitted to 49th IEEE Conference on Decision and Control (CDC '10). Available http://arxiv.org/abs/1004.2285.

[8] Laumanns M, Thiele L, Deb K, Zitzler E (2002) Combining convergence and diversity in evolutionary multiobjective optimization. Evolutionary Computation 10: 263-282.

[9] Newman MEJ (2002) Spread of epidemic disease on networks. Phys Rev E 66: 016128.

[10] Noël PA, Davoudi B, Brunham RC, Dubé LJ, Pourbohloul B (2009) Time evolution of epidemic disease on finite and infinite networks. Phys Rev E 79: 026101.

[11] Law A, Kelton WD (1999) Simulation Modeling and Analysis. New York: McGraw-Hill Higher Education, 3 edition.

[12] Colcombet T (2002) On families of graphs having a decidable first order theory with reachability. In: Diaz J, et al., editors, Automata, Languages and Programming: 29th International Colloquium, ICALP. pp. 98–109.

[13] Keeling MJ (1999) The effects of local spatial structure on epidemiological invasions. Proc R Soc Lond B 266: 859–867.

[14] Lindelauf RH, Borm PE, Hamers H (2009) The Influence of Secrecy on the Communication Structure of Covert Networks. Social Networks 31.

[15] Gutfraind A (2010). The extent of SIR epidemics on arbitrary graphs is monotonic. http://arxiv.org/abs/1005.3470. Submitted to Journal of Mathematical Biology.

[16] Draief M, Ganesh A, Massoulié L (2008) Thresholds for virus spread on networks. Annals of Applied Probability 18: 359-378.
